# Methodology of peri-operative imaging data reporting for stone disease: a systematic review toward the development of a novel checklist—iSTAR

**DOI:** 10.1007/s00345-026-06433-x

**Published:** 2026-06-11

**Authors:** Eugenia Vercelli, Mauro Van den Ende, Frederick Hellemans, Filip Poelaert, Kim Pauwaert, Eva Van Bos, Matthias Boeykens, Pieter De Visschere, Khurshid Ghani, Casey Dauw, Panagiotis Kallidonis, Thomas Tailly

**Affiliations:** 1 University of Turin, Turin, Italy; 2 Department of Urology, Molinette University Hospital – Città della Salute e della Scienza, Turin, Italy; 3 Department of Urology, Ghent University Hospital, Ghent, Belgium; 4 University of Antwerp, Antwerp, Belgium; 5 Department of Urology, ZAS, Antwerp, Belgium; 6 Department of Urology, AZ Groeninge, Kortrijk, Belgium; 7 Department of Radiology and Nuclear Medicine, Ghent University Hospital, Ghent, Belgium; 8 Department of Urology, University of Michigan, Ann Arbor, MI USA; 9 Department of Urology, University Hospital of Patras, Patras, Greece

**Keywords:** Imaging, Urolithiasis, Percutaneous nephrolithotomy, Ureteroscopy, Shockwave lithotripsy, Stone free rate

## Abstract

**Purpose:**

To critically analyze the methodology of imaging data reporting, including timing, modality and stone burden measurement perioperatively, from recently published literature on surgical interventions for stones, and to develop a scoring system to evaluate its quality.

**Methods:**

Articles reporting treatment outcomes in adult patients, published in 2020–2021 by preselected journals, were searched on PubMed and Embase. Recorded data for statistical analysis included type of study and intervention, pre- and postoperative imaging modality and timing, stone burden assessment method and measurement, stone free and treatment success definition or their differentiation. A new 7-point checklist (iSTAR—Imaging for Stone Treatment Assessment Reporting) was developed.

**Results:**

A total of 122 studies were included. Several papers did not report on pre- or postoperative imaging modality (13.9% and 5.7%), nor provided a definition of stone burden measurement or treatment success (43% and 18%). Stone burden was most often assessed with Non-Contrast Computed Tomography (54.9%) and reported as stone diameter (75.4%). Definitions of treatment success varied widely across literature, ranging from 0 mm to allowing 4 mm residual fragments. Preoperative imaging timing lacked in 95.9% of studies, while postoperative timing distribution was heterogeneously present. iSTAR scores resulted in 0/7 (0.8%), 1/7 (0.8%), 2/7 (1.6%), 3/7 (7.4%), 4/7 (16.4%), 5/7 (32%), 6/7 (38.5%) and 7/7 (10.7%), independently from study design.

**Conclusion:**

This research unveils a lack of standardization in reporting of perioperative urolithiasis imaging. iSTAR checklist may help authors to improve data reporting and provide journals and reviewers with a framework to assess imaging methodology quality in submitted manuscripts.

**Supplementary Information:**

The online version contains supplementary material available at 10.1007/s00345-026-06433-x.

## Purpose

The use of imaging for both diagnostic and therapeutic purposes in patients with urolithiasis has shown considerable variability in clinical practice, largely dependent on the discretion of healthcare providers. Current guidelines offer a near-universal diagnostic and therapeutic approach for the surgical treatment of ureteral and renal stones. Both the European Association of Urology (EAU) and the American Urological Association (AUA) recommend a Non-Contrast Computed Tomography (NCCT) as the gold standard for assessing stone burden, with treatment algorithms typically based on linear measurements of stone size [1, 2].

However, these guidelines do not provide specific recommendations on timing and method for evaluating the effectiveness of stone treatment and classification of treatment outcomes. A lack of consensus on post-operative imaging and reporting of results was identified by multiple authors more than a decade ago. Studies by Hyams et al. and Patel et al. have highlighted significant variation in the imaging modalities used for pre-operative stone burden assessment [3, 4]. In an effort to address this, Fulgham et al. proposed decision trees to guide the selection of imaging modalities for diagnosis and post-treatment follow-up, based on stone density and patient's symptoms [5]. Furthermore, Opondo et al. conducted a Delphi process including a panel of 85 experts to standardize post-operative outcome reporting following percutaneous nephrolithotomy (PCNL) [6]. This panel identified post-operative CT imaging on either postoperative day 1 or after one month as the preferred method, and ‘the absence of residual stone fragments’ as the optimal definition of being "stone free" (SF). A similar effort was made by International Alliance of Urolithiasis (IAU) panel, recommending a clinical framework for PCNL in the attempt to comprehensively standardize the procedure peri-operatively [7]. Despite this, there remains no clear consensus as to what constitutes a Clinically Insignificant Residual Fragment (CIRF).

While Fulgham’s patient-, stone-, and treatment-tailored approach offers a framework for clinical practice, the considerable variation in imaging modalities, timing of follow-up, and definitions of success hampers the comparison of outcomes across studies [5]. This heterogeneity also complicates the pooling of data for meta-analyses in stone-related research, as highlighted by Mazzon et al. [8] Moreover, imaging plays a key role in the search for standardization in the stone disease treatment, so imaging data reporting is of the utmost importance. Considering these issues, the objective of this paper is to critically review the recently published literature regarding the use and reporting of imaging modalities before and after intervention for urolithiasis, as well as the definitions employed for stone burden assessment and treatment outcomes. Additionally, we aimed to develop a scoring system to evaluate the quality of stone burden imaging reporting in current literature.

## Methods

**Fig. 1:**
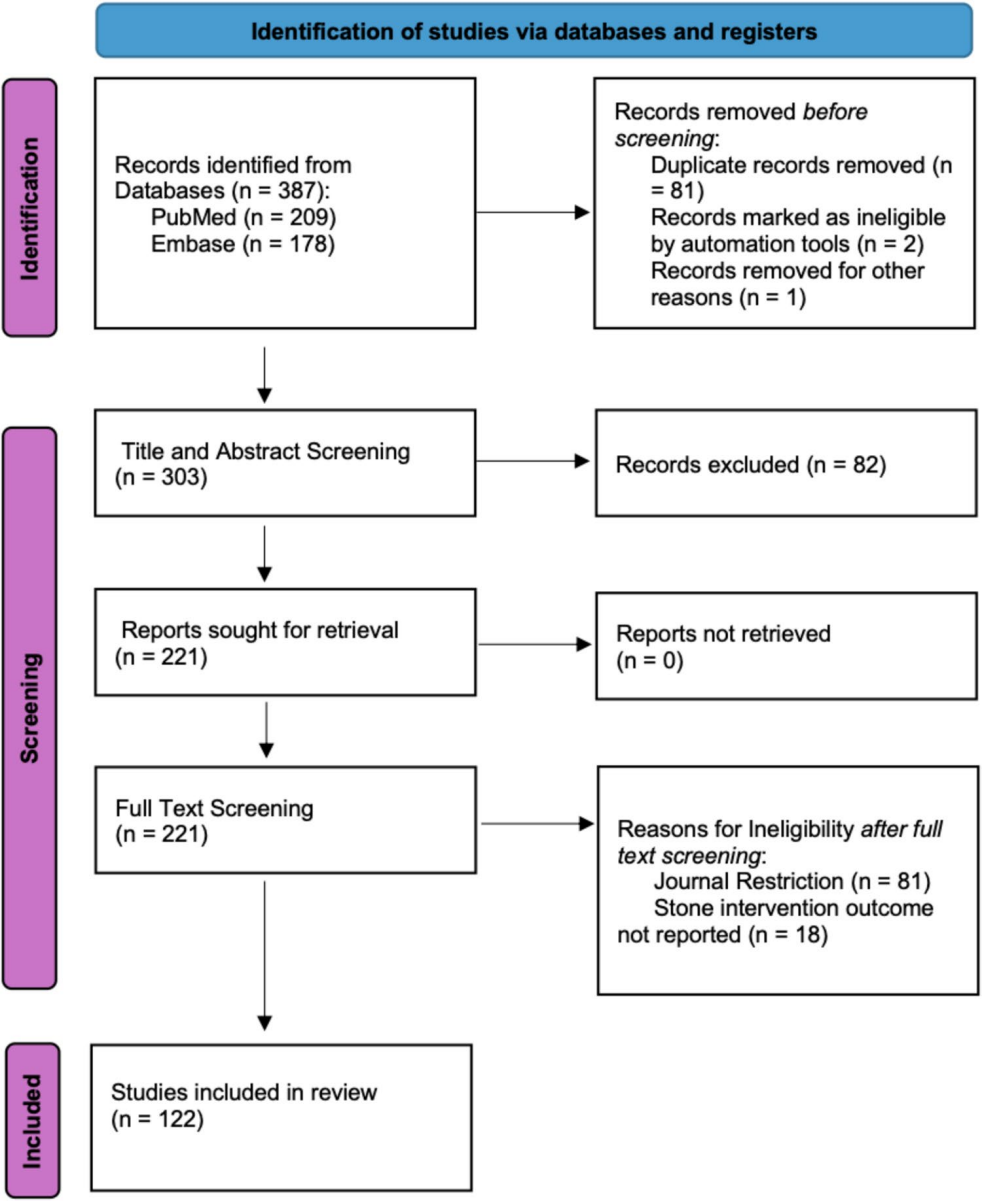
Prisma flowchart of study selection according to the 2020 PRISMA checklist

The present systematic review was conducted following the Preferred Reporting Items for Systematic Reviews and Meta-Analyses (PRISMA) guidelines, adhering to the 2020 PRISMA checklist [9] (Fig. 1).

We conducted a search of PubMed and Embase databases for literature published in 2020 and 2021. The search was limited to a pre-selected list of urological journals, depending on their impact factor at the time and the rate of published articles on the theme: *BJU International*, *European Urology (Focus)*, *Journal of Endourology*, *Journal of Urology*, *Urolithiasis*, *Urology*, and *World Journal of Urology*. English language restriction was applied. Search keywords were: “kidney stones,” “ureteral stones,” “renal stones,” “urolithiasis,” “nephrolithiasis,” “ureterolithiasis,” “ureteroscopy,” “shockwave lithotripsy,” and “percutaneous nephrolithotomy.” Studies were considered eligible if they reported outcomes of stone interventions in adult patients. Exclusion criteria were systematic reviews, meta-analyses, letters to the editor, and abstracts without a full-text article. Two reviewers (EV and MVdE) independently screened the titles and abstracts. Discrepancies were resolved by a third reviewer (TT).

Following full-text screening, a data extraction sheet was developed. The following data were collected: first author, journal name, country of study, year of publication, duration of data collection (in months), study type, intervention type, imaging modality used for preoperative and postoperative evaluation, method of stone burden assessment (one dimension: maximal diameter; two dimensions: surface area; or three dimensions: volume), method of stone burden measurement, timing of pre- and post-operative imaging, and the definitions of “stone-free” and/or “treatment success” with distinctions made, if reported. Descriptive statistical analysis was performed using SPSS Statistics (version 29.0, IBM, Chicago, IL, USA). Qualitative variables were expressed as percentages, and quantitative variables as means. Mann–Whitney U test and Kruskal–Wallis test were conducted to compare retrospective studies to prospective studies, and each of those to randomized controlled trials (RCTs), respectively.

To assess the quality of reporting on the use of peri-operative imaging for stone treatment, we developed a novel scoring system: the iSTAR (Imaging for Stone Treatment Assessment Reporting) checklist. It provides a score ranging from 0 to 7, with 7 representing the highest quality of reporting. The score evaluates seven key domains related to imaging use in the literature: the presence of both pre- and post-operative imaging, the reported timing of pre- and post-operative imaging, the method of pre-operative stone measurement and the reporting of a definition for SF status and/or treatment success. Recognizing that treatment is often considered successful even in the presence of CIRF, we introduced a ‘B’ variable to the scoring system, differentiating manuscripts where both SF status and treatment success were explicitly differentiated (Table 1). The 7-item checklist was created through the structured process of extracting all imaging-related reporting elements from the included studies. These items were then refined in internal discussions among the author group. Final inclusion of the seven domains was reached by consensus. Two reviewers (EV and MvdE) independently applied the proposed 7-item checklist to all included articles, and discrepancies were solved through discussion with a third senior reviewer (TT). As this study represents the initial proposal phase of the checklist, a Delphi process for the development of the checklist was not used and a statistical assessment of interrater reliability (e.g., Cohen’s kappa) was not the focus of this study.

**Table 1:** Individual criteria for imaging for stone treatment assessment reporting checklist (iSTAR checklist)

Imaging for stone treatment assessment reporting checklist (iSTAR checklist)		Yes	No
(1)	Was pre-operative imaging modality reported?	1	0
(2)	Was pre-operative imaging timing reported?	1	0
(3)	Was pre-operative stone measurement reported?	1	0
(4)	Was a definition of stone burden measurement method reported?	1	0
(5)	Was post-operative imaging modality reported?	1	0
(6)	Was post-operative imaging timing reported?	1	0
(7)	Was a definition of “stone free” and/or “treatment success” reported?		
	A definition of “stone free” *or* “treatment success” was reported	1	0
	Definition of both “stone free” *and* “treatment success” were reported and differentiated from one another	B	–

## Results

A total of 122 studies were included, published predominantly in the *Journal of Endourology* (n = 42, 34.4%) and *World Journal of Urology* (n = 40, 32.8%), followed by *Urolithiasis* (n = 21, 17.2%), with the remaining journals contributing < 5% each. Of these, 68 studies (55.7%) were prospective (33 non-RCTs—27%; 35 RCTs – 28.7%), and 54 (44.3%) were retrospective. The studies reported on Ureterorenoscopy (URS) (30 studies; 24.9%), Extracorporeal Shockwave Lithotripsy (ESWL) (17 studies; 13.9%), PCNL (52 studies; 42.6%) and Endoscopic Combined Intrarenal Surgery (ECIRS) (1 study; 0.8%) as a single intervention. In 22 studies (18.0%), multiple interventions were reported.

The frequency of pre- and post-operative imaging modalities is summarized in Table 2. For pre-operative stone burden assessment, NCCT was the predominant imaging modality, utilized across all treatment types in 67 studies (54.9%). Notably, 17 studies (13.9%) failed to specify the pre-operative imaging modality used to assess stone burden. Multiple imaging modalities were reported in 30 studies (24.6%), with combinations of KUB (Kidney-Ureter-Bladder Radiography), ultrasound and NCCT. Stone diameter, a linear measurement of stone burden, was the most widely used metric in 92 studies (75.4%), while surface area and volume measurements were reported in only 25 studies (20.5%) and 23 studies (18.8%), respectively. Unfortunately, detailed descriptions of how stone burden was measured in one, two, or three dimensions were frequently missing, with 40.2%, 52%, and 21.7% of studies, respectively, lacking this information (Table 3). A wide variability was observed in both imaging modalities and parameters used for post-operative evaluation of stone clearance. Multiple imaging modalities were more frequently employed for post-operative follow-up (62 studies; 50.8%) and NCCT was used less frequently (38 studies; 31.1%).

**Table 2:** Peri-operative imaging modalities before and after different types of intervention, including separated imaging for stone treatment assessment reporting (iSTAR) scores per type of intervention

	Surgical treatment					Frequency
	ESWL only 17/122 (13.9%)	URS only 30/122 (24.6%)	PCNL only 52/122 (42.6%)	ECIRS only 1/122 (0.8%)	Multiple 22/122 (18%)	122/122 (100%)
Imaging modality (pre-operative)						
KUB only	1 (5.9%)*	0 (0%)*	0 (0%)*	0 (0%)*	0 (0%)*	1 (0.8%)**
US only	0 (0%)*	0 (0%)*	1 (1.9%)*	0 (0%)*	0 (0%)*	1 (0.8%)**
NCCT only	10 (58.8%)*	20 (66.7%)*	25 (48.1%)*	1 (100.0%)*	11 (50.0%)*	67 (54.9%)**
CECT only	0 (0%)*	0 (0%)*	6 (11.5%)*	0 (0%)*	0 (0%)*	6 (4.9%)**
Multiple	6 (35.3%)*	4 (13.3%)*	11 (21.2%)*	0 (0%)*	9 (41%)*	30 (24.6%)**
NR	0 (0%)*	6 (20.0%)*	9 (17.3%)*	0 (0.0%)*	2 (9%)*	17 (13.9%)**
Imaging modality (post-operative)						
KUB only	5 (29.4%)*	3 (10.0%)*	3 (5.8%)*	0 (0%)*	2 (9.1%)*	13 (10.7%)**
US only	0 (0%)*	0 (0%)*	0 (0%)*	0 (0%)*	1 (4.5%)*	1 (0.8%)**
NCCT only	2 (11.8%)*	11 (36.7%)*	18 (34.6%)*	1 (100.0%)*	6 (27.3%)*	38 (31.1%)**
CECT only	0 (0%)*	0 (0%)*	1 (1.9%)*	0 (0%)*	0 (0%)*	1 (0.8%)**
Multiple	10 (58.8%)*	12 (40.0%)*	29 (55.8%)*	0 (0%)*	11 (50.0%)*	62 (50.8%)**
NR	0 (0%)*	4 (13.3%)*	1 (1.9%)*	0 (0%)*	2 (9.1%)*	7 (5.7%)**
Stone burden***						
Diameter	13	23	37	1	18	92
Surface	1	1	20	0	3	25
Volume	6	5	10	0	2	23
NR	0	4	5	0	2	11
iSTAR score						
iSTAR 0	0 (0%)*	0 (0%)*	1 (1.9%)*	0 (0%)*	0 (0%)*	1 (0.8%)**
iSTAR 1	0 (0%)	1 (3.3%)*	0 (0%)*	0 (0%)*	0 (0%)*	1 (0.8%)**
iSTAR 2	0 (0%)	1 (3.3%)*	1 (1.9%)*	0 (0%)*	0 (0%)*	2 (1.6%)**
iSTAR 3	0 (0%)	3 (10%)*	4 (7.8%)*	0 (0%)*	2 (9.1%)*	9 (7.3%)**
iSTAR 4	0 (0%)	8 (26.7%)*	8 (15.4%)*	0 (0%)*	4 (18.2%)*	20 (16.4%)**
iSTAR 5	7 (41.2%)	7 (23.3%)*	15 (28.8%)*	1 (100%)*	9 (40.9%)*	39 (32.0%)**
iSTAR 6	10 (58.8%)	10 (33.4%)*	21 (40.4%)*	0 (0%)*	6 (27.3%)*	47 (38%)**
iSTAR 7	0 (0%)	0 (0%)*	2 (3.8%)*	0 (0%)*	1 (4.5%)*	3 (2.4%)**

*Individual percentages as proportion of all studies reporting on this specific intervention

**Total percentage as proportion of all 122 included studies

***Percentages of Stone Burden could not be provided as articles possible report on multiple stone burden measurements

**Table 3:** Pre-operative stone burden measurement reporting studies as stone diameter, stone area and stone volume with separated stone burden measurement methods

Pre-operative stone burden reporting			Frequency	%
Stone burden reported as	**Diameter**		**92**	**75.4**
	Measurement method reported	Yes	55	59.8
		No	37	40.2
	**Area**		**25**	**20.5**
	Measurement method reported	Yes	12	48.0
		No	13	52.0
	**Volume**		**23**	**18.8**
	Measurement method reported	Yes	18	78.3
		No	5	21.7
	**Not reported**		**11**	**9**

We observed considerable variation in the timing of post-operative imaging used to assess SF status, with a mean of 27-days post-procedure (range 1–90 days). In 43 studies (35.2%), imaging was performed within the first day to the seventh day post-operatively; 30 studies (24.6%) conducted imaging after discharge but within one month; and 29 studies (23.8%) performed imaging one month or later after the procedure. In 20 studies (16.4%) the timing of post-operative imaging was missing. More surprisingly, pre-operative imaging timing was not reported in 95.9% of the included articles.

Regarding the definition of treatment success or SF, many articles defined a threshold for CIRF considered acceptable for declaring a procedure successful. In 14 studies (11.5%), fragments < 2 mm were classified as CIRFs, while 49 studies (40.2%) set the threshold between 2 and 4 mm. However, 22 studies (18.0%) did not report any definition for SF or treatment success, despite reporting success rates. A total of 37 studies (30.3%) defined SF as the complete absence of stone fragments. The distribution of these definitions in relation to treatment modalities is detailed in Table 4. In contrast, only 50 studies (41.0%) provided a clear definition of treatment success. Of these, some included CIRF in their definitions, with cut-offs of 0 mm, 0–2 mm, or 2–4 mm reported in 16 studies (43.2%), 6 studies (42.9%), and 23 studies (46.9%), respectively. Among the 22 studies (18%) that did not provide a specific cut-off value for CIRF, 5 studies (22.7%) claimed to define treatment success but did not specify a CIRF definition (Table 4).

**Table 4:** Reported definition of treatment success and reported cut-off values for clinical insignificant residual fragments (CIRF) and stone free (SF), separated by CIRF cut-off value

		CIRF cut-off value				Total
		No CIRF	CIRF up to 2 mm	CIRF up to 4 mm	Not reported	
Definition of success	Yes	16 (43.2%)**	6 (42.9%)**	23 (46.9%)**	5 (22.7%)**	50 (41.0%)**
	No	21 (56.8%)**	8 (57.1%)**	26 (53.1%)**	17 (77.3%)**	72 (59.0%)**
Surgical treatment	ESWL	6 (35.2%)*	0 (0%)*	8 (47.0%)*	3 (17.6%)*	17 (13.9%)**
	URS	7 (23.3%)*	10 (33.3%)*	8 (26.6%)*	5 (16.6%)	30 (24.6%)**
	PCNL	16 (30.7%)*	3 (5.8%)*	25 (48.1%)*	8 (15.3%)*	52 (42.6%)**
	ECIRS	1 (100%)*	0 (0%)*	0 (0%)*	0 (0%)*	1 (0.8%)**
	Multiple/others	7 (31.8%)*	1 (4.5%)*	8 (36.4%)*	6 (27.3%)*	22 (18.0%)**
Frequency		37 (30.3%)**	14 (11.5%)**	49 (40.2%)**	22 (18.0%)**	122 (100.0%)**

*Individual percentages as proportion of all studies reporting on this specific intervention

**Total percentage as proportion of all 122 included studies

**Fig. 2:**
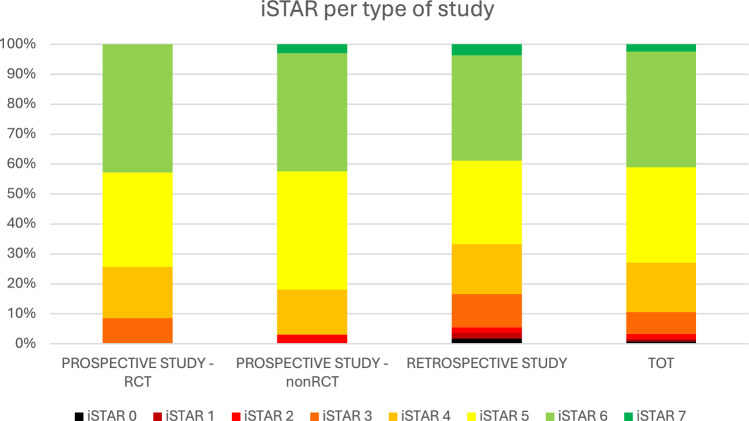
Application of iSTAR checklist on the articles included in our literature review, separated by type of study

Finally, the iSTAR checklist was applied to assess the quality of reporting across the included studies. The distribution of iSTAR scores is presented in Table 2. One study (0.8%) received a score of 0/7; 1 study (0.8%) scored 1/7; 2 studies (1.6%) scored 2/7; 9 studies (7.4%) scored 3/7; 22 studies (18%) scored 4/7; 39 studies (32.0%) scored 5/7; 47 studies (38.5%) scored 6/7, and 3 studies (2.5%) achieved the highest score of 7/7. Additionally, 13 studies (10.7%) received a 'B,' indicating that they provided separate definitions for both SF and treatment success (Table 2). When dividing iSTAR scores into groups depending on study design, the retrospective studies are scored with an iSTAR score of 4 or below more frequently than the prospective studies. Nevertheless, this difference is not statistically significant in the Mann–Whitney U test. When separating RCTs from other prospective studies and comparing to retrospective studies, no statistical differences could be found after applying the Kruskal–Wallis test (Fig. 2).

## Discussion

This study demonstrates significant variability in the use of pre- and post-operative imaging for evaluating stone burden in adult patients undergoing treatment for urolithiasis. While there is no direct evidence that variability in peri-operative imaging affects patient outcomes, the inconsistency in peri-operative imaging use and reporting hampers the ability to compare different treatment modalities and pool study results. A more standardized approach to peri-operative imaging is necessary to improve clinical decision-making and research outcomes.

### Pre-operative stone assessment

Since the 2010 report by Hyams et al., the use of NCCT for pre-operative stone assessment has increased significantly, from 27% to 54.9% [3]. In contrast, the use of KUB has dropped dramatically, from 60% to just one study in this review. This trend reflects the growing accessibility of NCCT and its ability to provide detailed imaging with lower radiation exposure due to the advantage of low-dose and ultra-low dose CT. Nevertheless, KUB, in combination with other modalities, remains in use for follow-up imaging likely due to its low cost and ease of access [10–12]. Despite the increased use of NCCT, the timing of pre-operative imaging is rarely reported —only 5 out of 122 manuscripts (4.1%) documented when imaging was performed. This lack of reporting introduces the potential for errors in the true assessment of stone burden, especially if significant time has passed between imaging and surgery. Accurate timing of pre-operative imaging is critical for determining the actual stone burden that needs to be treated, especially if the imaging was performed a considerable amount of time prior to the procedure.

Historically, stone diameter has been the most used measurement to assess pre-operative stone burden. Over the past decade, there has been an increased interest to use stone volume rather than linear measurements, as volume provides a more accurate representation of the total stone burden. Despite this shift in ideology, the use of stone diameter in reporting stone burden has remained largely unchanged (75.4% in Hyams et al. vs. 69% in the current review) [3]. Similarly, the use of surface area as a measurement has not changed significantly over time (20.5% vs. 21%). The use of stone volume, however, has increased from 4% to 18.8%, reflecting a growing recognition of its potential clinical importance. It should be emphasized that the iSTAR checklist is independent of the specific stone burden metric adopted, as its purpose is to assess the completeness and transparency of imaging data reporting rather than to promote a specific measurement method.

Notably, a large proportion of studies failed to clearly describe how exactly stone burden was measured—whether by diameter, surface area, or volume (40.2%, 52%, and 21.7%, respectively). This lack of important details makes it difficult to compare outcomes across studies. While guidelines generally recommend linear measurements, such as Cumulative Stone Diameter (CSD), our review confirms the increasing use of stone volume as a more accurate measure of stone burden. Panthier et al. recently supported this trend, demonstrating that automated stone volume measurement is the most precise method for assessing stone burden [13]. Although opponents to the use of stone volume might argue that CSD is easier and prompter to describe [14], a recent meta-analysis by the EAU guideline panel demonstrated that overall stone volume is a better predictor of SF status. This seems to count for ESWL and URS, whereas the data for PCNL was probably insufficient to reach any statistical significance. It should be nuanced that, although the areas under the ROC curves reached statistical significance, sufficient data to demonstrate the clinical impact of volume is still lacking and true superiority of volume as a predictor of the outcomes after stone treatment remains to be proven [15].

### Post-operative stone assessment and stone free

While NCCT has become more common for pre-operative imaging, its use for post-operative assessment of stone-free rate (SFR) has remained relatively stable compared to Hyams et al. (31.1% vs. 24%) [3]. However, there has been a decline in the use of KUB (10.7% vs. 75%) and ultrasound (0.8% vs. 44%) alone for post-operative imaging. This decline may be attributed to the well-documented limitations of these modalities in detecting small residual fragments, as KUB and ultrasound have lower sensitivity and specificity compared to CT [16].

The most recent EAU guidelines provide a consensus for follow-up imaging, depending on patients' stone-free status, risk of recurrence, and stone type [2]. For assessment of stone free status, imaging at four weeks after the intervention is suggested by the EAU guidelines to be most appropriate, while imaging modality remains at the surgeon’s discretion [17]. This lack of standardization prevents comparisons between different treatment modalities and underscores the need for a unified post-operative evaluation protocol. Notably, the *Journal of Endourology*, has made strides in this area by requiring CT as the preferred post-operative imaging modality. This policy shift is expected to have a significant impact on both clinical practice and future research [18].

Despite these efforts, real-world data suggest that post-operative imaging remains underutilized. Sutherland et al. reported that only 45% of patients in the United States underwent any post-operative imaging within three months of URS, and only 61% had imaging within one year [19]. Dauw et al. also found that over half of the patients in Michigan (52.4% out of 2850) did not receive any post-operative imaging after endoscopic stone treatment [20]. Among those who did, KUB was used in 55% of cases, while only 11.1% received CT imaging. These figures suggest that, despite existing recommendations, post-operative imaging protocols are not consistently followed in clinical practice and highlights a significant gap in patient care. The decision to use imaging often depends on factors such as stone burden, pre-operative stent placement, intraoperative endoscopic assessment of stone free status and patient comorbidities. Similar to long-term follow-up recommendations as published by the EAU guidelines, it is clear that a framework for the assessment of treatment success is also direly needed.

### Definitions of stone free and treatment success

Despite the recognized importance of post-operative imaging, there has been no significant improvement in the reporting of SF rate (SFR) or the definition of residual fragments (RF) in recent years. In our review, 32% of studies did not define SFR, a figure that is unchanged from Deters et al.'s findings over a decade ago [21]. While more studies today use RF < 4 mm as the cut-off for SF status (40.2% vs. 18%), only 30.3% of studies define SF as the complete absence of fragments, down from 47% in Deters et al. This inconsistency in defining SFR complicates comparisons between studies and limits our ability to draw meaningful conclusions from the current research.

The increasing use of CT for post-operative assessment, which is more likely to identify very small RF than a KUB and ultrasound, has complicated the interpretation of CIRF. Macejko et al. demonstrated that different SF definitions produce different outcomes: SFR was around 50% when defined as the absence of RF, 63% when RF < 2 mm, and 84% when RF < 4 mm [22]. While smaller RF are often considered acceptable, their long-term clinical significance remains unclear. The 2014 Delphi Consensus on PCNL emphasized the importance of distinguishing between SFR and treatment success [6]. Treatment success may include CIRF, but clear definitions are needed to guide clinical practice and research.

Similar to results obtained by the EAU-YAU (Young Academic Urologists) Endourology and Urolithiasis Working Group; our review identified 51% of PCNL studies that defined CIRF as fragments between 2 and 4 mm, while 71.4% of URS studies defined CIRF as 2 mm or smaller [23]. These differences suggest a threshold for "treatment success" varying on surgical approach. However, a consistent definition of treatment success is essential for comparing outcomes across different interventions. Chew et al. found that residual fragments between 2 and 4 mm are more likely to grow than those smaller than 2 mm, highlighting the need for a standardized approach to defining clinically significant residual fragments [24]. A minimal fragment size that necessitates reintervention should be agreed upon before considering a patient stone-free. The author guidelines of the Journal of Endourology in fact suggest reporting different grades of SF data as grade A, B and C for 0 mm, 2 mm and 4 mm RF.

Panthier et al. reinforced the importance of a standardized definition for CIRF, noting that it directly affects outcomes such as stone regrowth, emergency department visits, and reintervention rates [25]. Without a consistent timeframe and imaging modality for assessing SFR, these clinical outcomes are difficult to interpret across different studies.

Interestingly, while there appears to be an increasing interest in reporting pre-operative stone burden as volume, residual fragments are still consistently reported in a linear measurement. As mandated by the Journal of Endourology, if these would also be calculated as volumes, volume reduction may demonstrate to be a meaningful outcome parameter.

### iSTAR score

The iSTAR score is determined by a checklist of 7 items concerning pre- and postoperative imaging modality, timing and stone burden measurement. It represents an important step towards standardizing the reporting of imaging in the evaluation of stone disease in the perioperative setting. Of the studies included in this review, only three achieved a perfect iSTAR score of 7/7, while most scored between 5/7 and 6/7 (70.5%). The most common deficiencies were the reporting of pre-operative imaging timing and the method of stone burden measurement. The latter is especially concerning considering the increasing interest in identifying the most accurate way of measuring stone burden [26]. Interestingly, RCTs did not achieve significantly higher iSTAR scores than retrospective studies, highlighting the need for standardized guidelines in prospectively developed study designs. In fact, retrospective studies in our review achieved iSTAR scores comparable to prospective studies, suggesting that the prospective aspect of a study does not necessarily correlate with better methodology regarding peri-operative imaging (Fig. 2).

In 2014, Somani et al. attempted to develop a similar scoring system focused on post-operative imaging and SFR definitions [27]. However, the iSTAR checklist offers a more comprehensive evaluation of peri-operative imaging and will hopefully evolve into a valuable tool for improving the quality of reporting in future studies. Importantly, the iSTAR score should not be interpreted as a threshold for study validity or publication eligibility, but rather as a descriptive tool to identify strengths and gaps in peri-operative imaging reporting.

Similar to the longitudinal assessment of the quality of RCTs in the field of urolithiasis using the CONSORT score, this iSTAR scoring system can be applied in the same way [28]. Longitudinal bibliographical studies may identify an evolution in reporting quality of peri-operative imaging for urolithiasis.

### Limitations

Some limitations of this review should be acknowledged. The articles included were identified from only seven high-impact journals, published in a short timeframe between 2020 and 2021, which may limit the generalizability of our findings. Nevertheless, this time-restricted analysis provides a useful benchmark against which the impact of more recent reporting recommendations can be evaluated in future studies. As it was not within the aim of this manuscript, we did not collect dosage levels of CT or KUB imaging modalities if they had been published alongside in the manuscripts that were reviewed. This prevents us from comparing the use of low dose CT in comparison to regular dose CT or the evolution thereof over time in contrast to previous literature. Moreover, since the checklist is only at its inception, interrater reliability metrics have not been assessed, nor has the checklist been internally validated. Further research will focus on validating the applicability of iSTAR across different types of interventions and assess the reliability of the scoring across raters. In the longer term, we will explore its impact on the reporting of peri-operative imaging for stone surgery in published literature.

## Conclusion

The present study shows not only a lack of standardization, but also an extreme variability in reporting important details about peri-operative stone burden imaging assessment and stone treatment outcomes. The iSTAR scoring system was developed in an effort to quantify the completeness of reporting on peri-operative imaging for stone interventions. The checklist may help the authors to improve the quality of data reporting in future work and can provide journals and reviewers with a framework to assess imaging methodology quality in submitted manuscripts. Ultimately, this score could develop into a mandatory aspect of future stone research.

## Supplementary Information

Below is the link to the electronic supplementary material.

Supplementary file1 (DOCX 87 KB)

## Data Availability

No datasets were generated or analysed during the current study.

## Abbreviations

AUA: American urological association
CECT: Contrast-enhanced computed tomography
CIRF: Clinically insignificant residual fragments
EAU: European association of urology
ECIRS: Endoscopic combined intrarenal surgery
ESWL: Extracorporeal shock wave lithotripsy
iSTAR: Imaging for stone treatment assessment reporting
KUB: Kidney-ureter-bladder radiography
NCCT: Non-contrast computed tomography
NA: Not applicable
NR: Not reported
PCNL: Percutaneous nephrolithotomy
PRISMA: Preferred reporting items for systematic reviews and meta-analyses
RCT: Randomized controlled trial
RF: Residual fragments
SF: Stone-free
SFR: Stone-free rate
URS: Ureterorenoscopy
US: Ultrasound
