# Supplementary material for: Methodology of peri-operative imaging data reporting for stone disease: a systematic review toward the development of a novel checklist—iSTAR
Source: World J Urol. 2026 Jun 11;44(1):420. doi: 10.1007/s00345-026-06433-x (PMC13260150; doi:10.1007/s00345-026-06433-x)
Supplement: Supplementary file 1 — Supplementary file1 (DOCX 87 KB) [file 345_2026_6433_MOESM1_ESM.docx]

Supplementary Materials

**Supplementary Bibliography of studies included in the systematic review** (1–122)

1. Gerber RC, Best SL, Hedican SP, Nakada SY. Flexible Ureteroscopy as the New Standard for the Management of Renal Transplant Urolithiasis &lt;15 mm: A Single-Center Experience. J Endourol. 2021 Oct 1;35(10):1443–7.

2. Knoedler MA, Li S, Best SL, Hedican SP, Penniston KL, Nakada SY. Clinical Impact of the Institution of Moses Technology on Efficiency During Retrograde Ureteroscopy for Stone Disease: Single-Center Experience. J Endourol. 2022 Jan 1;36(1):65–70.

3. Mager R, Brauers C, Kurosch M, Dotzauer R, Borgmann H, Haferkamp A. Outcomes for Geriatric Urolithiasis Patients aged ≥80 Years Compared to Patients in Their Seventies. Eur Urol Focus. 2022 Jul;8(4):1103–9.

4. Schoenthaler M, Hein S, Wilhelm K, Pohlmann PF, Praus F, Walther T, et al. Feasibility of an Updated Randomised Controlled Trial on Surgical Urolithiasis Treatments: The Pilot Trial for the German Endoscopic versus Shock Wave Therapy Study (GESS). Eur Urol Focus. 2022 Jan;8(1):271–5.

5. Hirsch B, Abt D, Güsewell S, Langenauer J, Betschart P, Pratsinis M, et al. Outcome groups and a practical tool to predict success of shock wave lithotripsy in daily clinical routine. World J Urol. 2021 Mar 20;39(3):943–51.

6. Dauw CA, Swarna K, Qi J, Kim T, Leavitt D, Leese J, et al. Shockwave Lithotripsy Use in the State of Michigan: American Urological Association Guideline Adherence and Clinical Implications. Urology. 2020 Mar;137:38–44.

7. Manzo BO, Gómez F, Figueroa A, Sánchez HM, Leal M, Emiliani E, et al. A New Simplified Biplanar (0-90°) Fluoroscopic Puncture Technique for Percutaneous Nephrolithotomy. Reducing Fluoroscopy Without Ultrasound. Initial Experience and Outcomes. Urology. 2020 Jun;140:165–70.

8. Ulvik Ø, Harneshaug JR, Gjengstø P. What Do We Mean by “Stone Free,” and How Accurate Are Urologists in Predicting Stone-Free Status Following Ureteroscopy? J Endourol. 2021 Jul 1;35(7):961–6.

9. Gupta P, Choudhary GR, Pandey H, Madduri VKS, Singh M, Pallagani L. Air vs contrast pyelogram for initial puncture access in percutaneous nephrolithotomy: a randomized controlled trial. Urolithiasis. 2021 Jun 7;49(3):261–7.

10. Biswas K, Gupta SK, Tak GR, Ganpule AP, Sabnis RB, Desai MR. Comparison of STONE score, Guy’s stone score and Clinical Research Office of the Endourological Society (CROES) score as predictive tools for percutaneous nephrolithotomy outcome: a prospective study. BJU Int. 2020 Oct 4;126(4):494–501.

11. Yoshioka T, Ikenoue T, Hashimoto H, Otsuki H, Oeda T, Ishito N, et al. Development and validation of a prediction model for failed shockwave lithotripsy of upper urinary tract calculi using computed tomography information: the S3HoCKwave score. World J Urol. 2020 Dec 22;38(12):3267–73.

12. Sahan M, Sarilar O, Savun M, Caglar U, Erbin A, Ozgor F. Adopting for Supine Percutaneous Nephrolithotomy: Analyzing the Learning Curve of Tertiary Academic Center Urology Team. Urology. 2020 Jun;140:22–6.

13. Soliman T, Sherif H, Sebaey A, Mohey A, Elmohamady BN. Miniperc *vs* Shockwave Lithotripsy for Average-Sized, Radiopaque Lower Pole Calculi: A Prospective Randomized Study. J Endourol. 2021 Jun 1;35(6):896–901.

14. Pietropaolo A, Hendry J, Kyriakides R, Geraghty R, Jones P, Aboumarzouk O, et al. Outcomes of Elective Ureteroscopy for Ureteric Stones in Patients with Prior Urosepsis and Emergency Drainage: Prospective Study over 5 yr from a Tertiary Endourology Centre. Eur Urol Focus. 2020 Jan;6(1):151–6.

15. Tao R zhen, Tang Q lai, Zhou S, Jia C ping, Lv J lin. External physical vibration lithecbole facilitating the expulsion of upper ureteric stones 1.0–2.0 cm after extracorporeal shock wave lithotripsy: a prospective randomized trial. Urolithiasis. 2020 Feb 28;48(1):71–7.

16. Armas‐Phan M, Tzou DT, Bayne DB, Wiener S V., Stoller ML, Chi T. Ultrasound guidance can be used safely for renal tract dilatation during percutaneous nephrolithotomy. BJU Int. 2020 Feb 3;125(2):284–91.

17. Sugino Y, Kato T, Furuya S, Sasaki T, Arima K, Sugimura Y. The usefulness of the maximum Hounsfield units (HU) in predicting the shockwave lithotripsy outcome for ureteral stones and the proposal of novel indicators using the maximum HU. Urolithiasis. 2020 Feb 11;48(1):85–91.

18. Kim JJ, Suh YS, Han DH. Comparison of outcomes in totally tubeless percutaneous nephrolithotomy according to nephrostomy tract sealing with fibrin versus gelatin matrix: a propensity score matching study. Urolithiasis. 2020 Apr 14;48(2):151–8.

19. Elbaset MA, Hashem A, Eraky A, Badawy MA, El-Assmy A, Sheir KZ, et al. Optimal non-invasive treatment of 1–2.5 cm radiolucent renal stones: oral dissolution therapy, shock wave lithotripsy or combined treatment—a randomized controlled trial. World J Urol. 2020 Jan 3;38(1):207–12.

20. Lu P, Chen K, Wang Z, Song R, Zhang J, Liu B, et al. Clinical efficacy and safety of flexible ureteroscopic lithotripsy using 365 μm holmium laser for nephrolithiasis: a prospective, randomized, controlled trial. World J Urol. 2020 Feb 27;38(2):481–7.

21. Alotaibi KM. Retrograde nephrostomy access for percutaneous nephrolithotomy: a simple and safe technique. Urolithiasis. 2020 Apr 29;48(2):175–81.

22. Irer B, Sahin MO, Erbatu O, Yildiz A, Ongun S, Cinar O, et al. Impact of previous SWL on ureterorenoscopy outcomes and optimal timing for ureterorenoscopy after SWL failure in proximal ureteral stones. World J Urol. 2020 Mar 16;38(3):769–74.

23. Zhao Z, Sun H, Zeng T, Deng T, Liu Y, Zeng G. An easy risk stratification to recommend the optimal patients with 2–3 cm kidney stones to receive retrograde intrarenal surgery or mini-percutaneous nephrolithotomy. Urolithiasis. 2020 Apr 17;48(2):167–73.

24. Lima A, Reeves T, Geraghty R, Pietropaolo A, Whitehurst L, Somani BK. Impact of ureteral access sheath on renal stone treatment: prospective comparative non-randomised outcomes over a 7-year period. World J Urol. 2020 May 24;38(5):1329–33.

25. Qi S, Yang E, Bao J, Yang N, Guo H, Wang G, et al. Single-Use Versus Reusable Digital Flexible Ureteroscopes for the Treatment of Renal Calculi: A Prospective Multicenter Randomized Controlled Trial. J Endourol. 2020 Jan 1;34(1):18–24.

26. Yamashita S, Kohjimoto Y, Iguchi T, Nishizawa S, Kikkawa K, Hara I. Ureteral wall volume at ureteral stone site is a critical predictor for shock wave lithotripsy outcomes: comparison with ureteral wall thickness and area. Urolithiasis. 2020 Aug 23;48(4):361–8.

27. Wu X, Zhao Z, Sun H, Cai C, Li Z, Cheng D, et al. Day-surgery percutaneous nephrolithotomy: a high-volume center retrospective experience. World J Urol. 2020 May 14;38(5):1323–8.

28. Kandemir E, Savun M, Sezer A, Erbin A, Akbulut MF, Sarılar Ö. Comparison of Miniaturized Percutaneous Nephrolithotomy and Standard Percutaneous Nephrolithotomy in Secondary Patients: A Randomized Prospective Study. J Endourol. 2020 Jan 1;34(1):26–32.

29. Torricelli FCM, Monga M, Yamauchi FI, Marchini GS, Danilovic A, Vicentini FC, et al. Renal Stone Features Are More Important Than Renal Anatomy to Predict Shock Wave Lithotripsy Outcomes: Results from a Prospective Study with CT Follow-Up. J Endourol. 2020 Jan 1;34(1):63–7.

30. Agarwal DK, Krambeck AE, Sharma V, Maldonado FJ, Westerman ME, Knoedler JJ, et al. Treatment of non-obstructive, non-struvite urolithiasis is effective in treatment of recurrent urinary tract infections. World J Urol. 2020 Aug 23;38(8):2029–33.

31. Goldberg H, Nevo A, Shtabholtz Y, Lubin M, Baniel J, Margel D, et al. Tubeless supra‐costal percutaneous nephrolithotomy is associated with significantly less hydrothorax: a prospective randomized clinical study. BJU Int. 2020 Feb 3;125(2):276–83.

32. Khadgi S, EL-Nahas AR, Darrad M, AL-Terki A. Safety and efficacy of a single middle calyx access (MCA) in mini-PCNL. Urolithiasis. 2020 Dec 10;48(6):541–6.

33. Aminsharifi A, Irani D, Tayebi S, Jafari Kafash T, Shabanian T, Parsaei H. Predicting the Postoperative Outcome of Percutaneous Nephrolithotomy with Machine Learning System: Software Validation and Comparative Analysis with Guy’s Stone Score and the CROES Nomogram. J Endourol. 2020 Jun 1;34(6):692–9.

34. Reeves T, Pietropaolo A, Somani BK. Ureteroscopy and Laser Stone Fragmentation Is Safe and Tends to Improve Renal Function in Patients with Chronic Kidney Disease: Prospective Outcomes with a Minimum Follow-Up of 6 Months. J Endourol. 2020 Apr 1;34(4):423–8.

35. Lai D, Chen M, Sheng M, Liu Y, Xu G, He Y, et al. Use of a Novel Vacuum-Assisted Access Sheath in Minimally Invasive Percutaneous Nephrolithotomy: A Feasibility Study. J Endourol. 2020 Mar 1;34(3):339–44.

36. Xiong L, Huang X, Ye X, Chen L, Ma K, Liu J, et al. Microultrasonic Probe Combined with Ultrasound-Guided Minipercutaneous Nephrolithotomy in the Treatment of Upper Ureteral and Renal Stones: A Consecutive Cohort Study. J Endourol. 2020 Apr 1;34(4):429–33.

37. Dresner SL, Iremashvili V, Best SL, Hedican SP, Nakada SY. Influence of Lower Pole Infundibulopelvic Angle on Success of Retrograde Flexible Ureteroscopy and Laser Lithotripsy for the Treatment of Renal Stones. J Endourol. 2020 Jun 1;34(6):655–60.

38. Bakr M, Abdelhalim KM. Safety and Efficacy of Emergency Ureteroscopy with Intracorporeal Lithotripsy in Patients Presented with Urinary Tract Infection with Mild Sepsis. J Endourol. 2020 Mar 1;34(3):262–6.

39. Yamashita S, Iwahashi Y, Deguchi R, Kikkawa K, Kohjimoto Y, Hara I. Three-dimensional mean stone density on non-contrast computed tomography can predict ureteroscopic lithotripsy outcome in ureteral stone cases. Urolithiasis. 2020 Dec 28;48(6):547–52.

40. Tailly T, Nadeau BR, Violette PD, Bao Y, Amann J, Nott L, et al. Stone Burden Measurement by 3D Reconstruction on Noncontrast Computed Tomography Is Not a More Accurate Predictor of Stone-Free Rate After Percutaneous Nephrolithotomy Than 2D Stone Burden Measurements. J Endourol. 2020 May 1;34(5):550–7.

41. Mains EAA, Blackmur JP, Sharma AD, Gietzmann WK, El-Mokadem I, Stephenson C, et al. Shockwave Lithotripsy Is an Efficacious Treatment Modality for Obese Patients with Upper Ureteral Calculi: Logistic Regression and Matched-Pair Analyses from a Dedicated Center Comparing Treatment Outcomes by Skin-to-Stone Distance. J Endourol. 2020 Apr 1;34(4):487–94.

42. Al Adl AM, Mohey A, Abdel Aal A, Abu-Elnasr HAF, El Karamany T, Noureldin YA. Percutaneous Nephrolithotomy Outcomes Based on S.T.O.N.E., GUY, CROES, and S-ReSC Scoring Systems: The First Prospective Study. J Endourol. 2020 Dec 1;34(12):1223–8.

43. Enikeev D, Taratkin M, Klimov R, Alyaev Y, Rapoport L, Gazimiev M, et al. Thulium-fiber laser for lithotripsy: first clinical experience in percutaneous nephrolithotomy. World J Urol. 2020 Dec 27;38(12):3069–74.

44. Hegazy M, El-Assmy A, Ali-El-Dein B, Sheir KZ. The alternating bidirectional versus the standard approach during shock wave lithotripsy for upper lumbar ureteric stones: a randomized controlled trial. World J Urol. 2021 Jan 23;39(1):247–53.

45. Guler Y, Erbin A, Kafkasli A, Ozmerdiven G. Factors affecting success in the treatment of proximal ureteral stones larger than 1 cm with extracorporeal shockwave lithotripsy in adult patients. Urolithiasis. 2021 Feb 11;49(1):51–6.

46. Liu Y, Cai C, Aquino A, Al‐Mousawi S, Zhang X, Choong SKS, et al. Management of large renal stones with super‐mini percutaneous nephrolithotomy: an international multicentre comparative study. BJU Int. 2020 Jul 25;126(1):168–76.

47. Koc E, Kamaci D, Gok B, Bedir F, Metin BC, Atmaca AF. Does the renal parenchymal thickness affect the efficacy of the retrograde intrarenal surgery? A prospective cohort study. Urolithiasis. 2021 Feb 13;49(1):57–64.

48. Vicentini FC, Mazzucchi E, Gökçe Mİ, Sofer M, Tanidir Y, Sener TE, et al. Percutaneous Nephrolithotomy in Horseshoe Kidneys: Results of a Multicentric Study. J Endourol. 2021 Jul 1;35(7):979–84.

49. Fathelbab TK, Hasanein MGS, Fawzy AM. Anterior or posterior SWL in proximal ureteral stones opposite to 4th and 5th lumbar vertebrae? World J Urol. 2021 Jan 15;39(1):255–61.

50. Guven S, Yigit P, Tuncel A, Karabulut İ, Sahin S, Kilic O, et al. Retrograde intrarenal surgery of renal stones: a critical multi-aspect evaluation of the outcomes by the Turkish Academy of Urology Prospective Study Group (ACUP Study). World J Urol. 2021 Feb 28;39(2):549–54.

51. Zhu H, Zhao Z, Cheng D, Wu X, Yue G, Lei Y, et al. Multiple-tract percutaneous nephrolithotomy as a day surgery for the treatment of complex renal stones: an initial experience. World J Urol. 2021 Mar 23;39(3):921–7.

52. Yadav BK, Basnet RB, Shrestha A, Shrestha PM. Comparison between shockpulse and pneumatic lithotripsy in percutaneous nephrolithotomy. World J Urol. 2021 Mar 24;39(3):915–9.

53. Zhong W, Wen J, Peng L, Zeng G. Enhanced super-mini-PCNL (eSMP): low renal pelvic pressure and high stone removal efficiency in a prospective randomized controlled trial. World J Urol. 2021 Mar 26;39(3):929–34.

54. Kallidonis P, Vagionis A, Vrettos T, Adamou K, Pagonis K, Ntasiotis P, et al. Non papillary mini-percutaneous nephrolithotomy: early experience. World J Urol. 2021 Apr 29;39(4):1241–6.

55. Nestler S, Grüne B, Schilchegger L, Neisius A, Jones J. Evaluation of stone free rates in early versus delayed primary ureteroscopy: time does matter. World J Urol. 2021 Mar 2;39(3):909–14.

56. Ferreira TAC, Dutra MMG, Vicentini FC, Szwarc M, Mota PKV, Eisner B, et al. Impact of Obesity on Outcomes of Supine Percutaneous Nephrolithotomy. J Endourol. 2020 Dec 1;34(12):1219–22.

57. Zanetti SP, Lievore E, Fontana M, Turetti M, Gallioli A, Longo F, et al. Vacuum-assisted mini-percutaneous nephrolithotomy: a new perspective in fragments clearance and intrarenal pressure control. World J Urol. 2021 Jun 26;39(6):1717–23.

58. Guan W, Fan S, Liang J, Feng N, Liang Q, Huang Y, et al. Management of Migrated or Residual Stones Following Laparoscopic Pyelolithotomy and Ureterolithotomy in Abnormal Kidneys: A Prospective and Randomized Comparison. J Endourol. 2020 Nov 1;34(11):1155–60.

59. Gadelkareem RA, Abdelsalam YM, Ibraheim MA, Reda A, Sayed MAB, El-Azab AS. Is Percutaneous Nephrolithotomy the Modality of Choice Versus Extracorporeal Shockwave Lithotripsy for a 20 to 30 mm Single Renal Pelvic Stone with ≤1000 Hounsfield Unit in Adults? A Prospective Randomized Comparative Study. J Endourol. 2020 Nov 1;34(11):1141–8.

60. Van den Broeck T, Zhu X, Kusters A, Futterer J, Langenhuijsen J, d’Ancona F. Percutaneous Nephrolithotomy with Intraoperative Computed Tomography Scanning Improves Stone-Free Rates. J Endourol. 2021 Mar 1;35(3):267–73.

61. Zhao F, Li J, Tang L, Li C. A comparative study of endoscopic combined intrarenal surgery (ECIRS) in the galdakao-modified supine valdivia (GMSV) position and minimally invasive percutaneous nephrolithotomy for complex nephrolithiasis: a retrospective single-center study. Urolithiasis. 2021 Apr 10;49(2):161–6.

62. Zhao Z, Yin S, Zhu H, Cheng D, Liu Y, Zeng G. The feasibility of multiple-tract mini-percutaneous nephrolithotomy as an overnight surgery for the treatment of complex kidney stones. Urolithiasis. 2021 Apr 24;49(2):167–72.

63. Amaresh M, Hegde P, Chawla A, de la Rosette JJMCH, Laguna MP, Kriplani A. Safety and efficacy of superior calyceal access versus inferior calyceal access for pelvic and/or lower calyceal renal calculi- a prospective observational comparative study. World J Urol. 2021 Jun 31;39(6):2155–61.

64. Cui HW, Tan TK, Christiansen FE, Osther PJS, Turney BW. The utility of automated volume analysis of renal stones before and after shockwave lithotripsy treatment. Urolithiasis. 2021 Jun 14;49(3):219–26.

65. Sonmez G, Demir F, Keske M, Karadag MA, Demirtas A. Comparison of the Effects of Four Treatment Techniques Commonly Used in Ureteral Stone Treatment on Patients’ Daily Physical Functioning: An Observational Randomized-Controlled Study. J Endourol. 2021 Jan 1;35(1):8–13.

66. Guo X, Zhang Z, Liu Z, Fu H, Gao X, Yang H, et al. Assessment of the Contrast-Enhanced Ultrasound in Percutaneous Nephrolithotomy for the Treatment of Patients with Nondilated Collecting System. J Endourol. 2021 Apr 1;35(4):436–43.

67. Emiliani E, Piccirilli A, Cepeda-Delgado M, Kanashiro AK, Mantilla D, Amaya CA, et al. Flexible ureteroscopy in extreme elderly patients (80 years of age and older) is feasible and safe. World J Urol. 2021 Jul 22;39(7):2703–8.

68. Shah D, Patil A, Reddy N, Singh A, Ganpule A, Sabnis R, et al. A clinical experience of thulium fibre laser in miniperc to dust with suction: a new horizon. World J Urol. 2021 Jul 22;39(7):2727–32.

69. Komeya M, Odaka H, Watanabe T, Kiuchi H, Ogawa T, Yao M, et al. Gap between UAS and ureteroscope predicts renal stone-free rate after flexible ureteroscopy with the fragmentation technique. World J Urol. 2021 Jul 25;39(7):2733–9.

70. Zeng G, Cai C, Duan X, Xu X, Mao H, Li X, et al. Mini Percutaneous Nephrolithotomy Is a Noninferior Modality to Standard Percutaneous Nephrolithotomy for the Management of 20–40 mm Renal Calculi: A Multicenter Randomized Controlled Trial. Eur Urol. 2021 Jan;79(1):114–21.

71. Saita A, Villa L, Persico F, Lughezzani G, Prezioso D, Casale P. In-vitro and in-vivo new evidence for Flexor® VueTM deflecting endoscopic system use: optimization of the stone free rate (SFR) after flexible ureteroscopy and Ho:YAG laser lithotripsy. Urolithiasis. 2021 Jun 1;49(3):239–45.

72. Thakur A, Sharma AP, Devana SK, Parmar KM, Mavuduru RS, Bora GS, et al. Does Miniaturization Actually Decrease Bleeding After Percutaneous Nephrolithotomy? A Single-Center Randomized Trial. J Endourol. 2021 Apr 1;35(4):451–6.

73. Izol V, Deger M, Akdogan N, Ok F, Bayazit Y, Aridogan IA. The Effect of Percutaneous Nephrolithotomy on the Estimated Glomerular Filtration Rate in Patients with Chronic Kidney Disease. J Endourol. 2021 May 1;35(5):583–8.

74. Ozbek R, Senocak C, Haberal HB, Damar E, Sadioglu FE, Bozkurt OF. Comparison of scoring systems for predicting stone-free status and complications after retrograde ıntrarenal surgery. World J Urol. 2021 Jul 15;39(7):2741–6.

75. Atis G, Culpan M, Ucar T, Sendogan F, Kazan HO, Yildirim A. The effect of shock wave lithotripsy and retrograde intrarenal surgery on health-related quality of life in 10–20 mm renal stones: a prospective randomized pilot study. Urolithiasis. 2021 Jun 19;49(3):247–53.

76. Lievore E, Boeri L, Zanetti SP, Fulgheri I, Fontana M, Turetti M, et al. Clinical Comparison of Mini-Percutaneous Nephrolithotomy with Vacuum Cleaner Effect or with a Vacuum-Assisted Access Sheath: A Single-Center Experience. J Endourol. 2021 May 1;35(5):601–8.

77. Chung JH, Baek M, Park SS, Han DH. The Feasibility of Pop-Dusting Using High-Power Laser (2 J × 50 Hz) in Retrograde Intrarenal Surgery for Renal Stones: Retrospective Single-Center Experience. J Endourol. 2021 Mar 1;35(3):279–84.

78. Danilovic A, Torricelli FCM, Marchini GS, Batagello C, Vicentini FC, Traxer O, et al. Residual Stone Fragments After Percutaneous Nephrolithotomy: Shockwave Lithotripsy *vs* Retrograde Intrarenal Surgery. J Endourol. 2021 May 1;35(5):609–14.

79. Demir M, Dere O, Yağmur İ, Katı B, Pelit ES, Albayrak İH, et al. Usability of shear wave elastography to predict the success of extracorporeal shock-wave lithotripsy: prospective pilot study. Urolithiasis. 2021 Jun 26;49(3):255–60.

80. Ito K, Takahashi T, Kanno T, Okada T, Higashi Y, Yamada H. Decreased Recurrence of Urolithiasis After Simultaneous Ureteroscopic Surgery for Ureter and Ipsilateral Renal Calculi: Comparison to Shockwave Lithotripsy for Ureter Calculi Alone. Urology. 2021 Jan;147:74–80.

81. Selmi V, Sari S, Oztekin U, Caniklioglu M, Isikay L. External Validation and Comparison of Nephrolithometric Scoring Systems Predicting Outcomes of Retrograde Intrarenal Surgery. J Endourol. 2021 Jun 1;35(6):781–8.

82. Cornelius J, Zumbühl D, Afferi L, Mordasini L, Di Bona C, Zamboni S, et al. Immediate Shockwave Lithotripsy *vs* Delayed Shockwave Lithotripsy After Urgent Ureteral Stenting in Patients with Ureteral or Pyeloureteral Urolithiasis: A Matched-Pair Analysis. J Endourol. 2021 May 1;35(5):721–7.

83. Xiao B, Zhang G, Ji C, Jin S, Hu W, Bai W, et al. Percutaneous Nephrolithotomy Under X-Ray–Free Technique in Upper Urinary Stone Patients with Autosomal Dominant Polycystic Kidney Disease: Experience from a Large-Volume Stone Management Center. J Endourol. 2021 Jul 1;35(7):967–72.

84. Large T, Assmus MA, Valadon C, Emmott A, Forbes CM, Agarwal D, et al. A Multi-institutional Review of Single-access Percutaneous Nephrolithotomy for Complex Staghorn Stones. Eur Urol Focus. 2021 Sep;7(5):1170–5.

85. Falahatkar R, Shahraki T, Falahatkar S, Esmaeili S, Mashouf P. Evaluating outcomes of complete supine percutaneous nephrolithotomy for staghorn vs multiple non-staghorn renal stones: a 10-year study. World J Urol. 2021 Aug 5;39(8):3071–7.

86. Micali S, Sighinolfi MC, Iseppi A, Morini E, Calcagnile T, Benedetti M, et al. Initial Experience and Evaluation of a Nomogram for Outcome Prediction in Management of Medium-sized (1–2 cm) Kidney Stones. Eur Urol Focus. 2022 Jan;8(1):276–82.

87. Abdelbary AM, Al-Dessoukey AA, Moussa AS, Elmarakbi AA, Ragheb AM, Sayed O, et al. Value of early second session shock wave lithotripsy in treatment of upper ureteric stones compared to laser ureteroscopy. World J Urol. 2021 Aug 20;39(8):3089–93.

88. Fawzy AM, Abdelkafy AA, Elahawy MM, Abdelgawad AH, Hasanein MGS, Abdelgawad AT, et al. Contralateral Coupling During Extracorporeal Shockwave Lithotripsy for Stones in Ectopic Kidney: Is It Feasible? J Endourol. 2021 Jul 1;35(7):1090–6.

89. EL-Nahas AR, Khadgi S, Diab M, AL-Terki A. Definition and Unfavorable Risk Factors of Trifecta in Mini-Percutaneous Nephrolithotomy. J Endourol. 2021 Aug 1;35(8):1140–5.

90. Guliev B, Komyakov B, Talyshinskii A. Interior definition of the calyceal orientation suitable for percutaneous nephrolithotripsy via mobile software. Urolithiasis. 2021 Oct 12;49(5):443–9.

91. Batagello CA, Vicentini FC, Monga M, Miller AW, Marchini GS, Torricelli FCM, et al. Tranexamic acid in patients with complex stones undergoing percutaneous nephrolithotomy: a randomised, double‐blinded, placebo‐controlled trial. BJU Int. 2022 Jan 13;129(1):35–47.

92. Hameed BMZ, Shah M, Naik N, Singh Khanuja H, Paul R, Somani BK. Application of Artificial Intelligence-Based Classifiers to Predict the Outcome Measures and Stone-Free Status Following Percutaneous Nephrolithotomy for Staghorn Calculi: Cross-Validation of Data and Estimation of Accuracy. J Endourol. 2021 Sep 1;35(9):1307–13.

93. Göger YE, Özkent MS, Kılınç MT, Taşkapu HH, Göger E, Aydın A, et al. Efficiency of retrograde intrarenal surgery in lower pole stones: disposable flexible ureterorenoscope or reusable flexible ureterorenoscope? World J Urol. 2021 Sep 18;39(9):3643–50.

94. Tsaturyan A, Bellin A, Barbuto S, Zampakis P, Ntzanis E, Lattarulo M, et al. Technical aspects to maximize the hyperaccuracy three-dimensional (HA3DTM) computed tomography reconstruction for kidney stones surgery: a pilot study. Urolithiasis. 2021 Dec 3;49(6):559–66.

95. Yue G, Lei Y, Karagöz MA, Zhu H, Cheng D, Cai C, et al. Comparison of the Prone Split-Leg Position with the Traditional Prone Position in Percutaneous Nephrolithotomy: A Propensity Score-Matching Study. J Endourol. 2021 Sep 1;35(9):1333–9.

96. Large T, Nottingham C, Brinkman E, Agarwal D, Ferrero A, Sourial M, et al. Multi-Institutional Prospective Randomized Control Trial of Novel Intracorporeal Lithotripters: ShockPulse-SE *vs* Trilogy Trial. J Endourol. 2021 Sep 1;35(9):1326–32.

97. Meier K, Hiller S, Dauw C, Hollingsworth J, Kim T, Qi J, et al. Understanding Ureteral Access Sheath Use Within a Statewide Collaborative and Its Effect on Surgical and Clinical Outcomes. J Endourol. 2021 Sep 1;35(9):1340–7.

98. Agrawal S, Patil A, Sabnis RB, Singh AG, Ganpule AP, Desai MR. Initial experience with slimmest single-use flexible ureteroscope Uscope PU3033A (PUSENTM) in retrograde intrarenal surgery and its comparison with Uscope PU3022a: a single-center prospective study. World J Urol. 2021 Oct 10;39(10):3957–62.

99. Thakare N, Tanase F, Saeb-Parsy K, Atassi N, Endriss R, Kamphuis G, et al. Efficacy and safety of the EMS Swiss LithoClast® Trilogy for PCNL: results of the European multicentre prospective study on behalf of European Section of UroTechnology. World J Urol. 2021 Nov 15;39(11):4247–53.

100. Zhang LW, Fei X, Song Y. The clinical efficacy of novel vacuum suction ureteroscopic lithotripsy in the treatment of upper ureteral calculi. World J Urol. 2021 Nov 17;39(11):4261–5.

101. Ahmed A, Abdelazim H, ElMesery M, El‐feky M, Gomaa A, Tagreda I, et al. Mini‐percutaneous nephrolithotomy is a safe alternative to extracorporeal shockwave lithotripsy for high‐density, renal stones: a prospective, randomised trial. BJU Int. 2021 Dec 13;128(6):744–51.

102. El-Shaer W, Shaboob E, Abdel-Lateef S. Minipercutaneous Nephrolithotomy Under Mixture of Local Anesthesia: A Randomized Controlled Study. J Endourol. 2021 Dec 1;35(12):1750–6.

103. Chong JT, Dunne M, Magnan B, Abbott J, Davalos JG. Ambulatory Percutaneous Nephrolithotomy in a Free-Standing Surgery Center: An Analysis of 500 Consecutive Cases. J Endourol. 2021 Dec 1;35(12):1738–42.

104. Radfar MH, Nasiri M, Shemshaki H, Sarhangnejad R, Dadpour M. A study on comparative outcomes of totally ultrasonography-guided percutaneous nephrolithotomy in prone versus flank position: a randomized clinical trial. World J Urol. 2021 Nov 3;39(11):4241–6.

105. AbdelRazek M, Abolyosr A, AbdelKader MS, Hassan AM, Hamed AA, Alsagheer G. Percutaneous nephrolithotomy versus extracorporeal shock wave lithotripsy for renal insufficiency. World J Urol. 2021 Dec 2;39(12):4477–82.

106. Shabana W, Oquendo F, Hodhod A, Ahmad A, Alaref A, Trigo S, et al. Miniaturized Ambulatory Percutaneous Nephrolithotomy Versus Flexible Ureteroscopy in the Management of Lower Calyceal Renal Stones 10-20 mm: A Propensity Score Matching Analysis. Urology. 2021 Oct;156:65–70.

107. De Nunzio C, Ghahhari J, Lombardo R, Russo GI, Albano A, Franco A, et al. Development of a nomogram predicting the probability of stone free rate in patients with ureteral stones eligible for semi-rigid primary laser uretero-litothripsy. World J Urol. 2021 Nov 26;39(11):4267–74.

108. Ghazi A, Melnyk R, Farooq S, Bell A, Holler T, Saba P, et al. Validity of a patient-specific percutaneous nephrolithotomy (PCNL) simulated surgical rehearsal platform: impact on patient and surgical outcomes. World J Urol. 2022 Mar 24;40(3):627–37.

109. Liaw CW, Khusid JA, Gallante B, Bamberger JN, Atallah WM, Gupta M. The T-Tilt Position: A Novel Modified Patient Position to Improve Stone-Free Rates in Retrograde Intrarenal Surgery. Journal of Urology. 2021 Nov;206(5):1232–9.

110. Yang E, Jing S, Niu Y, Qi S, Yadav PK, Yang L, et al. Single-Use Digital Flexible Ureteroscopes as a Safe and Effective Choice for the Treatment of Lower Pole Renal Stones: Secondary Analysis of a Randomized-Controlled Trial. J Endourol. 2021 Dec 1;35(12):1773–8.

111. Elbaset MA, Taha DE, Anas M, Abouelkheir RT, Edwan M, Abdullateef M, et al. Optimization of shockwave lithotripsy use for single medium sized hard renal stone with stone density ≥ 1000 HU. A prospective study. World J Urol. 2022 Jan 15;40(1):243–50.

112. Peretti D, Dalmasso E, Pecoraro A, Ambruosi C, Venzano F, Fiori C, et al. Low-energy high-frequency Ho-YAG lithotripsy: is RIRS going forward? A case–control study. Urolithiasis. 2022 Feb 23;50(1):79–85.

113. Kobayashi M, Waseda Y, Fuse H, Takazawa R. Variables measured on three-dimensional computed tomography are preferred for predicting the outcomes of shock wave lithotripsy. World J Urol. 2022 Feb 23;40(2):569–75.

114. Perrella R, Vicentini FC, Paro ED, Torricelli FCM, Marchini GS, Danilovic A, et al. Supine versus Prone Percutaneous Nephrolithotomy for Complex Stones: A Multicenter Randomized Controlled Trial. Journal of Urology. 2022 Mar;207(3):647–56.

115. García Rojo E, Teoh JYC, Castellani D, Brime Menéndez R, Tanidir Y, Galosi AB, et al. Real-world Global Outcomes of Retrograde Intrarenal Surgery in Anomalous Kidneys: A High Volume International Multicenter Study. Urology. 2022 Jan;159:41–7.

116. Pillai SB, Chawla A, de la Rosette J, Laguna P, Guddeti R, Reddy SJ, et al. Super-mini percutaneous nephrolithotomy (SMP) vs retrograde intrarenal surgery (RIRS) in the management of renal calculi ≤ 2 cm: a propensity matched study. World J Urol. 2022 Feb 12;40(2):553–62.

117. Liu X, Xia D, Peng E, Tong Y, Liu H, Wang X, et al. Comparison of two techniques for the management of 2–3 cm lower pole renal calculi in obese patients. World J Urol. 2022 Feb 12;40(2):513–8.

118. Fayad MK, Fahmy O, Abulazayem KM, Salama NM. Retrograde intrarenal surgery versus percutaneous nephrolithotomy for treatment of renal pelvic stone more than 2 centimeters: a prospective randomized controlled trial. Urolithiasis. 2022 Feb 22;50(1):113–7.

119. Patil A, Sharma R, Shah D, Gupta A, Singh A, Ganpule A, et al. A prospective comparative study of mini-PCNL using TrilogyTM or thulium fibre laser with suction. World J Urol. 2022 Feb 28;40(2):539–43.

120. Wu B, Bai S, Liu X. Combination laparoscopy and nephrolithotomy technique in the same session in patients with complete staghorn stones and poor performance status: case series in a single center with long-term follow-up. World J Urol. 2022 Mar 1;40(3):795–800.

121. Tsaturyan A, Lattarulo M, Adamou C, Pagonis K, Peteinaris A, Liourdi D, et al. The use of ureteral access sheath during mini-percutaneous nephrolithotomy with high-power holmium YAG laser. World J Urol. 2022 Mar 2;40(3):789–94.

122. Rezakahn Khajeh N, Majdalany SE, Ghani KR. Moses 2.0 for High-Power Ureteroscopic Stone Dusting: Clinical Principles for Step-by-Step Video Technique. J Endourol. 2021 Dec 1;35(S3):S-22-S-28.
